# Blood Pressure Variability and Outcome Predictors for Traumatic Brain Injury Patients with Diffuse Axonal Injury: A Retrospective Cohort Study

**DOI:** 10.5811/westjem.20346

**Published:** 2024-12-31

**Authors:** Christine E. Ren, Anastasia Ternovskaia, Fatima Mikdashi, Hassan Syed, Isha Vashee, Vainavi Gambhir, Natalie Chao, Jessica V. Downing, David Dreizin, Quincy K. Tran

**Affiliations:** * R. Adams Cowley Shock Trauma Center, University of Maryland Medical Center, Department of Emergency Medicine-Surgical Critical Care, Baltimore, Maryland; † Oregon Health and Science University, Department of Emergency Medicine and Critical Care Medicine, Portland, Oregon; ‡ University of Maryland School of Medicine, Department of Emergency Medicine, Research Associate Program, Baltimore, Maryland; § University of Maryland School of Medicine, Baltimore, Maryland; ∥ University of Maryland School of Medicine, Department of Emergency Medicine, Baltimore, Maryland; ¶ University of Maryland School of Medicine, Department of Diagnostic Radiology and Nuclear Imaging, Division of Emergency and Trauma Imaging, Baltimore, Maryland; # R. Adams Cowley Shock Trauma Center, University of Maryland Medical Center, Program in Trauma, Baltimore, Maryland

## Abstract

**Background:**

Diffuse axonal injury (DAI), a feature seen in severe traumatic brain injury (TBI), is associated with substantial morbidity and mortality. Although blood pressure variability (BPV) has been shown to impact TBI outcomes overall, its relevance in DAI cases remains uncertain. We investigated whether 24-hour post-injury BPV and other clinical factors were linked to patient outcomes.

**Methods:**

We conducted a retrospective analysis of Level I trauma center-admitted TBI patients with radiographic DAI diagnosis (computed tomography/magnetic resonance imaging). Hospital disposition (home, nursing facility, hospice/death) and Glasgow Coma Scale (GCS) on hospital day 5 (HD5GCS) were outcomes of interest. We assessed associations with clinical factors using ordinal logistic regression.

**Results:**

Among 153 patients (mean age 49 ±20 years, 74% male), median admission GCS was 5.0 (3.0-12.5), HD5GCS was 8.0 (6.0-11), and median hospital stay was 25 (15.5-34.5) days. The BPV, measured as successive variation in systolic blood pressure (SBP_SV_) and standard deviation in systolic blood pressure (SBP_SD_), was not significantly associated with hospital disposition. SBP_SV_ and SBP_SD_ were also not associated with our secondary outcome of HD5GCS. Initial international normalized ratio (INR) (Coefficient -3.67, odds ratio [OR] 0.03, 95% confidence interval [CI] 0.00-0.70), cerebral contusion (Coeff -2.39, OR 0.09, 95% CI 0.01-0.75), and HD5GCS (Coeff 0.59, OR 1.80, 95% CI 1.30-2.49) were associated with increased odds of discharge to hospice or death. Administration of blood products (Coeff 1.06, OR 2.89, 95% CI 1.10-7.60), vasopressors (Coeff 1.40, OR 4.05, 95% CI 1.37-11.96), and hyperosmolar therapy (Coeff 1.23, OR 3.41, 95% CI 1.36-8.54), and concurrent intraventricular hemorrhage (Coeff 0.99, OR 2.70, 95% CI 0.86-6.49) were linked to poorer HD5GCS.

**Conclusion:**

Blood pressure variability was not correlated with outcomes in patients with diffuse axonal injury. Low Glasgow Coma Score on hospital day 5, high initial INR, and concomitant cerebral contusion were associated with poorer outcomes.

Population Health Research Capsule What do we already know about this issue?

*Blood pressure variability (BPV) has been associated with poorer outcomes in patients with traumatic brain injury (TBI).*

What was the research question?

*Is BPV associated with worse disposition outcomes in TBI patients with diffuse axonal injury (DAI)?*

What was the major finding of the study?

*For TBI patients with DAI, blood pressure variability did not impact discharge destination.*

How does this improve population health?

*While BPV was not associated with poorer outcomes in our study, further studies are needed to determine whether other interventions can impact outcomes in these patients.*

## BACKGROUND

Diffuse axonal injury (DAI), also referred to as traumatic axonal injury, is an increasingly recognized component of traumatic brain injury (TBI), now estimated to occur in over 40% of patients hospitalized with other forms of TBI.^1^ ^,^ ^2^ DAI is caused by rotational acceleration-deceleration inertial forces that shear the white matter tracts in the brain. This mechanism disrupts axonal transport, leading to axonal swelling, secondary axonal disconnection, and subsequent degeneration.^3^ This injury is most often associated with high-velocity events, such as motor vehicle collisions or long falls from height. Clinical manifestations can vary widely, ranging from minimal significance to profound neurological impairment, depending on injury severity.

Severe cases of DAI often lead to persistent comas or substantial deficits in neurological recovery and are associated with significant mortality.^4^ Lesions associated with DAI may not initially be apparent or detectable on computed tomography (CT); patients with suspected DAI—often due to persistence of poor mental status in the absence of significant edema on CT or following neurosurgical evacuation of extra-axial hematoma—are often evaluated with magnetic resonance imaging (MRI) for diagnosis.^4^ ^,^ ^5^ The severity of DAI appreciated on MRI is characterized according to the degree and location of identified white matter lesions; Grade 1 is primarily associated with lesions in the cortex, Grade 2 in the corpus callosum, and Grade 3 in the brainstem.^6^

Given the variability in clinical manifestations and potentially high rates of cognitive morbidity and mortality associated with DAI, numerous studies have sought to identify features associated with improved or poor patient outcomes, such as radiographic findings, initial Glasgow Coma Scale (GCS) scores, and hypertension (defined as systolic blood pressure [SBP] ≥160 millimeters of mercury (mm Hg), among others.^4^ ^,^ ^7^ ^–^ ^9^ The role of blood pressure variability (BPV) has not yet been investigated in patients with DAI; BPV describes oscillations in blood pressure between consecutive measurements or within a defined timeframe. Variations in blood pressure are common after TBI, possibly due to impaired cerebrovascular autoregulation or decreased “baroreflex sensitivity” as a result of the injury, and prior research suggests a link to poor outcomes.^10^

Blood pressure variability has been associated with deviations from optimal cerebral perfusion pressures (CPPopt), which are in turn linked to unfavorable outcomes in TBI patients.^11^ We have previously investigated the connection between BPV and outcomes in patients with traumatic intraparenchymal hemorrhage and found an association with lower rates of discharge to home, indicating worse functional outcomes upon discharge.^12^ In addition to TBI, BPV has been previously associated with adverse outcomes in ischemic cerebrovascular accidents and spontaneous intracranial hemorrhage (ICH).^13^ ^–^ ^17^

In this study we investigated the impact of BPV in the initial 24 hours following hospital arrival on outcomes in patients with DAI and evaluated clinical features that may correlate with patient outcomes, with the goal of improving the accuracy of prognostic assessments and providing important information to guide future strategies in managing post-injury TBI and patients diagnosed with or suspected of having DAI.

## METHODS

### Study Setting

This study was performed at R. Adams Cowley Shock Trauma Center, a regional, quaternary trauma center and neurotrauma specialty center that admits trauma patients directly from the field and acts as a referral center for other hospitals within the state. Upon arrival at our institution, patients are first evaluated by the trauma team and undergo appropriate screening imaging studies, including CT, as clinically indicated. Patients with identified ICH or contusion are evaluated emergently by the neurosurgery team. Patients with CT or clinical characteristics suggestive of DAI subsequently undergo a brain MRI for confirmation and further characterization of disease severity when they are clinically stable enough to tolerate MRI. Previous studies have identified that radiographic presence of DAI on MRI is itself independently associated with poor outcomes^7^ ^,^ ^18^; thus, we chose patients who also had an MRI performed during their acute hospitalization within 30 days from their admission even if DAI was suspected on their initial CT images. This approach allowed us to better evaluate specific radiographic features such as hemorrhagic volume of burden and lesion location.

### Study Design, Patient Selection, and Data Collection

We conducted a retrospective cohort study of all adult trauma patients (≥18 years old) admitted to our hospital between January 1, 2016–December 31, 2019 with the diagnosis of TBI. Patients with radiographic evidence of DAI who underwent both CT and MRI within 30 days of admission were eligible. We excluded patients who did not have complete clinical information or imaging studies. Patients with a radiographic diagnosis of DAI were identified from our institution’s Radiology Information System, a database used for the management of radiographic images; further data was collected from the patient’s electronic health record (EHR).

Data abstraction followed previously published methodological guidelines on retrospective chart review.^19^ Prior to data collection, investigators evaluated sets of five patient charts and directly compared their findings to those of the senior investigator and principal investigator (Q.T.) until accuracy reached 90%. Data collectors were not blinded to the hypothesis. Radiographic information was interpreted and provided by an attending radiologist. An Excel spreadsheet (Microsoft Corporation, Redmond, WA) with standardized categories was used to record clinical data from de-identified patients.

Demographics and clinical data of interest, selected a priori according to a previous study,^18^ included the following: patient’s age; sex; past medical history; serum lactate level; international normalized ratio (INR); mechanism of injury; initial GCS at admission and highest recorded GCS at hospital day 5 (HD5GCS); administration of blood products (packed red blood cells, fresh frozen plasma, platelets, cryoprecipitate); vasopressors (norepinephrine, vasopressin, or epinephrine are the most commonly used vasopressors for this patient population at our institution); hyperosmolar therapy (hypertonic saline or mannitol); intravenous (IV) antihypertensives, antiepileptic medications, location and volume of DAI burden; concurrent presentation with seizures, intracranial contusion, intracerebral hemorrhage, intraventricular hemorrhage (IVH), or subarachnoid hemorrhage (SAH); and all recorded SBP measurements within the first 24 hours of admission. For patients who left the hospital or expired before hospital day 5, their HD5GCS levels were input as 3 (for expired patients) or the last recorded GCS prior to hospital discharge.

### Blood Pressure Variability

All blood pressure measurements were collected as they were recorded in patients’ charts by nursing staff. Our institution’s clinical standard dictates that patients admitted to intensive care units have at least one set of vital signs documented per hour. We collected all blood pressure measurements, as documented by our nursing staff, even if they exceeded more than one set of vital signs per hour. Methodology of obtaining blood pressure, either by manual blood pressure cuff, automatic blood pressure cuff, or by arterial blood pressure monitoring (radial or femoral access) was decided by the bedside clinicians. At our institution, invasive monitoring with arterial blood pressure monitoring is strongly encouraged for all patients who receive antihypertensives or vasopressor infusions. For patients who had documentation of both arterial blood pressure and cuff pressure, we collected the arterial blood pressure values.

Blood pressure variability quantifies blood pressure fluctuations over a specified time interval. The BPV can be studied with respect to SBP, diastolic blood pressure, and mean arterial pressure (MAP). Here, we examined variability in SBP, as specific SBP goals are traditionally used for management of patients with ICH or TBI.^20^ We evaluated three different modalities of measuring and reporting systolic BPV: successive variation of systolic blood pressure (SBP_SV_); standard deviation in systolic blood pressure (SBP_SD_); and coefficient of variation in systolic blood pressure (SBP_CV_).^21^ We also collected SBP_max_ and SBP_min_ from the first 24 hours of admission. The SBP_SV_ is the square root of the averaged squared difference between any two successive SBP measurements and demonstrates the rate of change between consecutive measurements. The SBP_SD_ represents the extent of variation or dispersion of individual SBP measurements around the average SBP within a given timeframe, indicating the level of fluctuation or stability in blood pressure values. The SBP_CV_ is calculated as the ratio of the standard deviation of SBP to the mean SBP and offers a standardized measure of SBP variability relative to the average SBP.

### Imaging Analysis

The presence of DAI was established based on MRI findings, which were interpreted and documented by an attending radiologist. The imaging information provided included the location of DAI within seven regions: the corpus callosum; basal ganglia; thalami; parahippocampal region; cerebellum; brainstem; and gray-white junction. The volume of DAI hemorrhage burden noted on susceptibility weighted images was measured in each location using the 3D slicer version 4.10 2 (https://www.slicer.org) sphere brush paint tool and quantification module. Additionally, presence or absence of concomitant injuries, specifically contusion, SAH, IVH, and intraparenchymal hemorrhage, were documented using radiology reports.

### Outcomes

Our primary outcome was hospital discharge disposition, used as a surrogate marker for neurocognitive disability at discharge among patients with TBI. Discharge destinations included home, rehabilitation facilities, and hospice/death. Being discharged home directly from the hospital signifies a favorable outcome with a higher likelihood of functional recovery and preservation of independent living. On the other hand, being discharged to a rehabilitation facility suggests the need for ongoing support and therapy due to significant neurologic deficits.^22^ Hospice/death represents the poorest outcome. The secondary outcome of HD5GCS has been shown to have prognostic value in predicting long-term outcomes and is considered an important indicator of neurological recovery in patients with spontaneous ICH.^23^ ^,^ ^24^

### Statistical Analysis

We used descriptive statistics to present continuous data as mean (standard deviation) or median (interquartile range), depending on the distribution of the data after the data’s histograms were inspected. The *t*-test or Mann-Whitney U test was employed for continuous data comparisons, while categorical data comparisons were conducted using the chi-square test or Fisher exact test, as appropriate. We used ordinal logistic regressions for the outcomes of both hospital disposition and HD5GCS. Hospital disposition was ranked in three orders from lowest to highest severity: 0 (home); 1 (rehabilitation); and 2 (hospice/death). Patients’ HD5GCS scores were ranked in order from 0 (GCS 3-8), 1 (GCS 9-12), 2 (GCS 13-14), or 3 (GCS 15). For the ordinal logistic regressions, the coefficients represent the association of the independent variables and the outcomes. A positive coefficient indicated increased odds of association with the lowest number rank (rank 0), while a negative coefficient was associated with the highest rank of the outcomes.

We performed all descriptive analyses and ordinal regressions with Minitab version 19 (Minitab LLC, State College, PA). All analyses with 2-tail *P* = < 0.05 were considered statistically significant.

## RESULTS

### Patient Characteristics

From the initial 174 patients identified in the EHR fitting our inclusion criteria, we included 153 in the final analysis. The remaining 23 patients were excluded due to inadequate recording of blood pressure, laboratory, or other clinical data (Figure). The mean age of included patients was 49 years (SD 20), and 113 (74%) were male (Table 1). Motor vehicle collisions were the most common mechanism of injury, accounting for 66% of the patients’ cause of injuries. Median GCS at admission was 5 (3-13). Among the study population, 141 patients (92%) required mechanical ventilation during their stay, and 94 patients (61%) underwent a tracheostomy procedure. The most common location of DAI burden was the corpus callosum (58%) followed by the parahippocampus (35%), basal ganglia (27%) and thalami (25%) (Table 1). Within the first 24 hours, all patients had received some form of opioid medication, 98% of the patients received IV fluids (IVF), 95% received a sedative medication, 87% received an anti-epileptic medication, and 82% required vasopressor support (Table 2).

**Figure.**
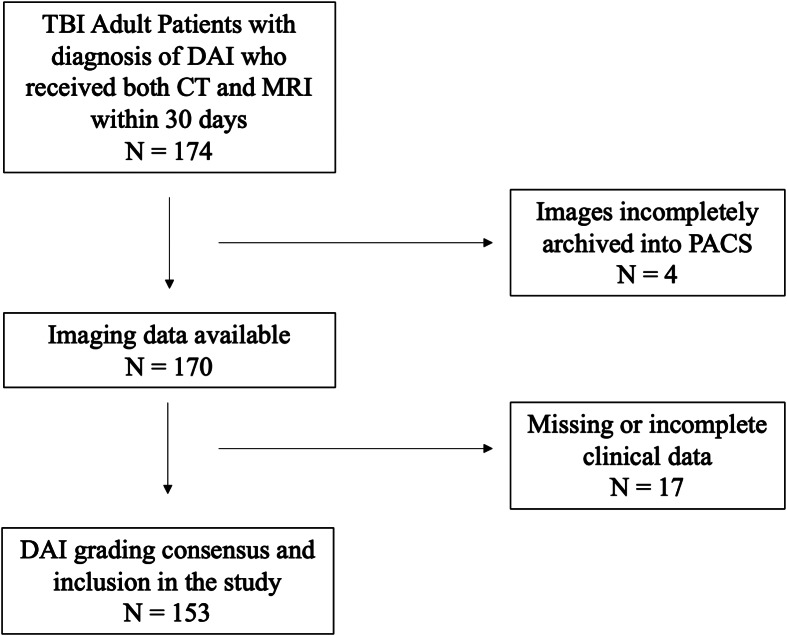
Flow diagram for patient selection. *TBI*, traumatic brain injury; *CT*, computed tomography; *MRI*, magnetic resonance imaging; *PACS*, picture archiving and communication system.

**Table 1.** Demographics and clinical features of patients with diffuse axonal injuries for the outcome of mortality.

	All patients N = 153	Alive N = 135	Dead/hospice N = 18	*P*-value
Age, years, mean (SD)	49 (20)	47 (19)	63 (19)	**0.002**
Sex, N (%)				
Male	113 (74)	99 (73)	14 (78)	0.67
Female	40 (26)	36 (27)	4 (22)	0.67
Past medical history, N (%)				
Hypertension	75 (49)	67 (50)	8 (44)	0.68
Diabetes	17 (11)	16 (12)	1 (6)	0.30
Clinical variables				
Hospital lengths of stay (days), median [IQR]	25.0 [15.5–34.5]	26.2 [17.9–37.1]	13.3 [8.3–17.0]	**<0.001**
GCS at admission, median [IQR]	5 [3–12]	6 [3–13]	4 [3–7]	0.16
GCS at 24 hours, median [IQR]	7 [5–10]	7 [6–15]	5 [4–6]	**<0.001**
GCS at 5 days, median [IQR]	8 [6–11]	9 [6–11]	5 [4–6]	**<0.001**
GCS 3–8, N (%)	83 (54)	67 (50)	16 (89)	**<0.001**
GCS 9–12, N (%)	41 (27)	40 (30)	1 (6)	**<0.001**
GCS 13–14, N (%)	14 (9)	14 (10)	0 (0)	**<0.001**
GCS 15, N (%)	14 (9)	14 (10)	0 (0)	**<0.002**
Admission lactate, mean (SD) (mg/dL)	3.8 (2.4)	4.0 (2.4)	3.0 (1.4)	**0.01**
Admission INR, mean (SD)	1.2 (0.3)	1.2 (0.18)	1.4 (0.6)	0.11
Mechanical ventilation, N (%)	141 (92)	123 (91)	18 (100)	**<0.001**
Tracheostomy, N (%)	94 (61)	92 (68)	2 (11)	**<0.001**
EVD, N (%)	57 (37)	51 (38)	6 (33)	0.71
Any IPH, N (%)	39 (25)	33 (24)	6 (33)	0.45
Any IVH, N (%)	93 (61)	76 (56)	17 (94)	**<0.001**
Any SAH, N (%)	125 (82)	111 (82)	14 (78)	0.67
Any contusion, N (%)	129 (84)	111 (82)	18 (100)	**<0.001**
Any seizure during hospitalization, N (%)	25 (16)	23 (17)	2 (11)	0.46
Any pneumonia	24 (16)	21 (16)	3 (17)	0.91
Any ARDS, N (%)	74 (48)	67 (50)	7 (39)	0.38
Mechanism of injury N (%)				
MVC	101 (66)	91 (67)	10 (56)	0.34
Fall	26 (17)	22 (16)	4 (22)	0.57
Penetrating trauma	1 (0.6)	1 (0.7)	0 (0)	0.32
Other blunt trauma	25 (16)	21 (16)	4 (22)	0.52
Location of injury, N (%)				
Corpus callosum	88 (58)	74 (55)	14 (78)	**0.03**
Basal ganglia	41 (27)	39 (29)	2 (11)	**0.03**
Thalami	38 (25)	35 (26)	3 (17)	0.33
Parahippocampus	53 (35)	47 (35)	6 (33)	0.90
Cerebellum	32 (21)	28 (21)	4 (22)	0.89
Brainstem	68 (44)	59 (44)	9 (50)	0.62
Volume of burden by location of injury, (mm^3^) median [IQR]				
Gray-white matter junction	1.4 [0.5–4.8]	1.4 [0.5–4.2]	1.7 [ 0.17–9.5]	0.73
Corpus callosum	0.02 [0–0.2]	0.02 [ 0–0.2]	0.2 [0.003–0.5]	**0.04**
Basal ganglia	0 [0–0.01]	0 [0–0.03]	0 [0–0]	0.17
Thalami	0 [0–0]	0 [0–0]	0 [0–0.5]	0.57
Parahippocampus	0 [0–0.03]	0 [0–0.03]	0 [0–0.2]	0.89
Cerebellum	0 [0–0]	0 [0–0]	0 [0–0.03]	0.75
Brainstem	0 [0–0.1]	0 [0–0.04]	0.01 [0–0.3]	0.30

Bolded values indicate statistical significance.

*CI*, confidence interval; *IQR*, interquartile range; *GCS*, Glasgow Coma Score; *mg*, milligram; *dL*, deciliter; *INR*, international normalized ratio; *EVD*, external ventricular drain; *IPH*, intraparenchymal hemorrhage; *IVH*, intraventricular hemorrhage; *SAH*, subarachnoid hemorrhage; *ARDS*, acute respiratory distress syndrome; *MVC*, motor vehicle collision; *mm*, millimeter.

**Table 2.** Clinical features within 24 hours for patients with diffuse axonal injuries.

	All patients	Alive	Dead/hospice	
	N = 153	n = 135	n = 18	*P*-value
Clinical interventions N (%)				
Any IVF	150 (98)	132 (98)	18 (100)	0.08
Any anti-seizure medication	133 (87)	118 (87)	15 (83)	0.66
Any hyperosmolar therapy	85 (56)	73 (54)	12 (67)	0.29
Any opioid	153 (100)	135 (100)	18 (100)	1.00
Any sedative	146 (95)	128 (95)	18 (100)	0.01
Any antihypertensive	102 (67)	88 (65)	14 (78)	0.24
Any vasopressor	123 (82)	108 (80)	15 (83)	0.72
Any tracheostomy	94 (61)	92 (68)	2 (11)	<0.001
Any blood products N (%)				
Platelets	19 (12)	15 (11)	4 (22)	0.27
PRBC	103 (67)	89 (66)	14 (78)	0.26
FFP	34 (22)	27 (20)	7 (39)	0.12
Cryoprecipitate	4 (3)	3 (2)	1 (6)	0.55
No blood products	103 (67)	95 (70)	8 (44)	0.04
Blood pressure variability				
SBP_max_, mean (SD)	166 (40)	166 (39)	169 (53)	0.77
SBP_min_, mean (SD)	97 (27)	97 (27)	92 (32)	0.52
SBP_max-min_, mean (SD)	69 (35)	68 (33)	77 (43)	0.41
SBP_SV_, mean (SD)	20.2 (9.4)	20.0 (9.4)	21.8 (9.2)	0.45
SBP_SD_, mean (SD)	20.3 (8.8)	20.1 (8.9)	21.8 (8.2)	0.42
SBP_CV_, mean (SD)	0.15 (0.07)	0.15 (0.07)	0.16 (0.01)	0.76
Hospital disposition, N (%)				
Home	12 (8)	12 (9)	0 (0)	<0.001
Nursing facility	123 (80)	123 (91)	0 (0)	<0.001

*DAI*, diffuse axonal injury; *IVF*, intravenous fluid; *PRBC*, packed red blood cells; *FFP*, fresh frozen plasma; *SBP*, systolic blood pressure; *max*, maximum; *min*, minimum; *SBP*_*SV*_, systolic blood pressure successive variation; *SBP*_*SD*_, systolic blood pressure standard deviation; *SBP*_*CV*_, systolic blood pressure coefficient of variation.

### Primary Outcome: Hospital Disposition

Our analysis identified no significant association between two separate measurements of BPV– SBP_SV_ (Coefficient -0.02, OR 0.98, 95% CI 0.87-1.10, *P* = 0.74) and SBP_SD_ (Coeff 0.03, OR 0.97, 95% CI 0.81-1.16, *P* = 0.74)–and hospital disposition among patients admitted for TBI and diagnosed with DAI. We found that 11.8% of patients with DAI either died in the hospital or were discharged to hospice care. This group of patients had a higher mean age of 63 (19) and lower GCS scores at 24 hours and 5 days (5 [4-6.3] for both) than survivors (Table 1). All these patients required intubation, all were diagnosed with a concurrent brain contusion, and 94% had a concurrent IVH. The corpus callosum was identified as the predominant location of DAI among patients who died, and a higher volume of hematoma was observed in the corpus callosum of this group when compared to survivors (Table 1). No significant differences were found in terms of sex, past medical history of hypertension and diabetes, or mechanism of injury.

We used ordinal logistic regression analysis to investigate the relationship between demographic and clinical factors and the likelihood of a significant discrepancy in the primary outcome of disposition (Table 3). The SBP variation measurements did not demonstrate an association with the disposition outcome (Table 4). Other clinical factors such as age, contusions, GCS scores, basal ganglia involvement, and the presence of SAH were found to be associated with discharge destination.

**Table 3.** Results from ordinal logistic regression assessing association between patients’ demographic and clinical factors and patients’ disposition, where order of hospital disposition was ranked from 0 = home, 1 = acute rehab, to 2 = hospice/death. All independent variables reported in this table were added in the model.

Variables	OR	95% CI	*P*-value	Coefficient
Age	0.97	0.93–1.00	**0.05**	−0.03
Sex: female	0.88	0.20–3.86	0.86	−0.13
Past medical history				
Hypertension	3.47	0.80–14.97	0.10	1.24
Diabetes	0.49	0.05–4.78	0.49	−0.72
Clinical factors				
Initial lactate	1.21	0.89–1.66	0.23	0.19
Initial INR	0.03	0.00–0.70	**0.03**	−3.67
Any blood products	0.36	0.08–1.58	0.18	−1.02
Any vasopressors	0.58	0.11–2.95	0.51	−0.54
Any hyperosmolar	0.75	0.17–3.33	0.71	−0.28
Any anti-hypertensives	0.22	0.04–1.06	0.06	−1.53
Any seizure	2.72	0.40–18.68	0.31	1.00
Any contusion	0.09	0.01–0.75	**0.03**	−2.39
Any AED	3.73	0.49–28.60	0.21	1.32
GCS at 5 days	1.80	1.30–2.49	**<0.001**	0.59
Location and burden of injury				
Corpus callosum	0.38	0.08–1.75	0.22	−0.96
Basal ganglia	5.02	1.02–24.62	**0.05**	1.61
Thalami	0.25	0.05–1.34	0.11	−1.37
Parahippocampus	1.27	0.26–6.26	0.77	0.24
Cerebellum	0.89	0.18–4.40	0.88	−0.12
Brainstem	0.71	0.18–2.82	0.63	−0.34
Any SAH	7.26	1.14–46.42	**0.04**	1.98
Any IVH	0.35	0.08–1.60	0.18	−1.05
Any IPH	2.94	0.68–12.80	0.15	1.08

Bolded *P*-values indicate statistical significance.

*OR*, odds ratio; *CI*, confidence interval; *GCS*, Glasgow Coma Score; *INR*, international normalized ratio; *AED*, antiepileptic drugs; *IPH*, intraparenchymal hemorrhage; *IVH*, intraventricular hemorrhage; *SAH*, subarachnoid hemorrhage.

**Table 4.** Results from ordinal logistic regression assessing association between blood pressure variability and patients’ disposition, where order of hospital disposition was ranked from 0 = home, 1 = acute rehab, to 2 = hospice/death.

Blood pressure variability	OR	95% CI	*P*-value	Coefficient
SBP_max_	1.00	0.96–1.04	0.92	−0.002
SBP_min_	1.00	0.96–1.05	0.92	0.002
SBP_SV_	0.98	0.87–1.10	0.74	−0.02
SBP_SD_	0.97	0.81–1.16	0.74	−0.03

*OR*, odds ratio; *CI*, confidence interval; *SBP*, systolic blood pressure; *SBP*_*SV*_, systolic blood pressure successive variation; *SBP*_*SD*_, systolic blood pressure standard deviation; *SBP*_*CV*_, systolic blood pressure coefficient of variation.

Among the demographic factors, age demonstrated a marginal association with the outcome (OR 0.97, 95% CI 0.93-1.00, *P* = 0.05, Coeff -0.03), suggesting that younger patients may be more likely to achieve favorable outcomes in terms of disposition. The presence of any cerebral contusion (OR 0.09, 95% CI 0.01-0.75, *P* = 0.03, Coeff -2.39) and higher initial INR (OR 0.03, 95% CI 0.00-0.70, *P* = 0.03, Coeff -3.67) correlated with poor disposition outcomes. These negative coefficients indicate that if there is contusion present or the value of the initial INR increases, the association with higher outcome numbers strengthens; in this case the highest outcome number is hospice/death.

Additionally, we identified GCS at five days as a significant factor affecting the outcomes of disposition (OR 95% CI 1.30-2.49, *P* = < 0.001, Coeff 0.59). Higher GCS scores at five days were strongly associated with an increased probability of achieving more favorable outcomes, such as discharge to home or rehabilitation. Regarding the location of burden, patients with involvement of the basal ganglia had poorer prognosis (OR 5.02, 95% CI 1.02-24.62, *P* = 0.05, Coeff 1.61). The presence of SAH was unexpectedly identified with better disposition outcomes (OR 7.26, 95% CI 1.14-46.42, *P* = 0.04, Coeff 1.98).

### Secondary Outcome: GCS at Hospital Day 5

The SBP_SV_ (Coeff 0.02, OR 1.02, 95% CI 0.95-1.1, *P* = 0.51) and SBP_SD_ (Coeff 0.02, OR 1.02, 95% CI 0.91-1.13, *P* = 0.75) were not associated with our secondary outcome of HD5GCS (Table 5). Receiving any blood products (OR 2.89, 95% CI 1.10-7.60, *P* = 0.03, Coeff 1.06), as well as treatment with vasopressors (OR 4.05, 95% CI 1.37-11.96, *P* = 0.01, Coeff 1.40), hyperosmolar therapy (OR 3.41, 95% CI 1.36-8.54, *P* = 0.01, Coeff 1.23), and the presence of concurrent IVH (OR 2.70, 95% CI 0.86-6.49, *P* = 0.03, Coeff 0.99) were all associated with an increased likelihood of a lower HD5GCS (Table 6). On the other hand, the use of antiepileptic drugs (OR 0.27, 95% CI 0.07-0.99, *P* = 0.05, Coeff -1.31) was associated with an increased likelihood of a higher HD5GCS.

**Table 5.** Results from ordinal logistic regression assessing association between blood pressure variability and patients’ hospital day five Glasgow Coma Score (GCS), which was ranked in order from 0 (GCS 3-8), 1 (GCS 9-12), 2 (GCS 13-14), 3 (GCS 15).

Blood pressure variability	OR	95% CI	*P*-value	Coefficient
SBP_max_	0.99	0.96–1.02	0.38	−0.01
SBP_min_	1.01	0.98–1.04	0.41	0.01
SBP_SV_	1.02	0.95–1.10	0.51	0.02
SBP_SD_	1.02	0.91–1.13	0.75	0.02

*GCS*, Glasgow Coma Score; *OR*, odds ratio; *CI*, confidence interval; *SBP*, systolic blood pressure; *SBP*_*SV*_, systolic blood pressure successive variation; *SBP*_*SD*_, systolic blood pressure standard deviation; *SBP*_*CV*_, systolic blood pressure coefficient of variation.

**Table 6.** Results from ordinal logistic regression assessing association between patients’ demographic and clinical factors and patients’ hospital day five Glasgow Coma Score (GCS), which was ranked in order from 0 (GCS 3-8), 1 (GCS 9-12), 2 (GCS 13-14), 3 (GCS 15). All independent variables reported in this table were added in the model.

Variables	OR	95% CI	*P*-value	Coefficient
Age	1.01	0.99–1.04	0.27	0.01
Sex: female	1.39	0.54–3.57	0.50	0.33
Past medical history				
Hypertension	0.74	0.29–1.88	0.53	−0.30
Diabetes	1.06	0.23–5.02	0.94	0.06
Clinical factors				
Initial lactate	1.00	0.84–1.19	0.98	0.003
Initial INR	3.80	0.58–24.93	0.16	1.33
Any blood products	2.89	1.10–7.60	**0.03**	1.06
Any vasopressors	4.05	1.37–11.96	**0.01**	1.40
Any hyperosmolar	3.41	1.36–8.54	**0.01**	1.23
Any anti-hypertensives	2.36	0.94–5.93	0.07	0.86
Any seizure	0.42	0.14–1.29	0.13	−0.86
Any contusion	1.18	0.36–3.88	0.78	0.17
Any AED	0.27	0.07–0.99	**0.05**	−1.31
Location of burden				
Corpus callosum	1.13	0.42–3.06	0.80	0.13
Basal ganglia	0.81	0.27–2.40	0.70	−0.21
Thalami	1.17	0.36–3.73	0.80	0.15
Parahippocampus	1.28	0.45–3.63	0.65	0.24
Cerebellum	1.03	0.33–3.21	0.96	0.03
Brainstem	3.24	1.33–7.86	**0.01**	1.17
Gray-white matter junction	0.39	0.01–17.59	0.63	−0.93
Any SAH	2.58	0.86–7.73	0.09	0.95
Any IVH	2.70	0.86–6.49	**0.03**	0.99
Any IPH	1.47	0.52–4.18	0.47	0.39

Bolded *P*-values indicate statistical significance.

*GCS*, Glasgow Coma Score; *OR*, odds ratio; *CI*, confidence interval; I*NR*, international normalized ratio; *AED*, antiepileptic drugs; *IPH*, intraparenchymal hemorrhage; *IVH*, intraventricular hemorrhage; *SAH*, subarachnoid hemorrhage.

## DISCUSSION

In this study we investigated the impact of 24-hour systolic BPV on outcomes in patients diagnosed with DAI and sought to identify relevant clinical features that may correlate with patient outcomes to improve prognostic assessments. We did not find a significant association between BPV and outcomes in patients with DAI. This stands in contrast to prior studies, such as that by Svedung Wettervik et al, who linked BPV to deviations from optimal CPPopt and unfavorable outcomes in patients with TBI.^25^ It has been proposed that the negative impact of BPV on patient outcomes may be attributed to the development of compromised cerebral blood flow regulation in TBI and the potential for secondary injuries such as cerebral hypoperfusion or hyperemia; however, the exact pathways and underlying processes are not fully understood.^26^ ^,^ ^27^ There are also several nuances, such as the duration and frequency of BPV monitoring, the timing of BPV in relation to the onset of injury, and the sensitivity of different BPV parameters, such as diastolic blood pressure or MAP variability, that require additional investigation and may also play a role in predicting outcomes.^28^ ^,^ ^29^

It is also unknown what role blood pressure management might play in mitigating the impacts of BPV. It is standard practice at our institution to manage hypertension (defined at the time of this study as SBP >160 mm Hg for patients with TBI) and hypotension (MAP <65 mm Hg) in patients with TBI using titratable infusions of antihypertensives and vasopressors. Strict management of blood pressure may have dampened BPV and limited our ability to detect an effect on patient outcomes. Lastly, BPV may have no impact on improving the damage caused by axonal shearing in DAI, or in preventing secondary axotomy. Additional studies are needed to clarify the interplay between BPV, cerebral hemodynamics, and DAI pathology, as well as to determine the most relevant and sensitive BPV parameters for predicting outcomes.

Our study revealed several clinical factors correlating with patient outcomes, specifically increased initial INR, concurrent cerebral contusion, and low GCS at hospital day 5. Our identification of initial INR as a poor prognostic factor with respect to hospital disposition contributes to the growing body of evidence on the association between coagulopathy and poor clinical outcomes in patients with TBI, specifically those with DAI.^30^ ^,^ ^31^ The disturbance in coagulation status at admission may exacerbate bleeding and contribute to a poorer prognosis. Coagulopathy induced by TBI follows a distinct pathogenic pathway, separate from coagulopathy induced by extracranial trauma and hemorrhagic shock. It can involve disruptions in the blood-brain barrier, which allow leakage of fluid and release procoagulant substances. These substances may also accelerate and enhance fibrinolysis. Early monitoring and management of coagulation abnormalities hold the potential to improve patient survival and reduce rates of mortality.^30^

Concurrent cerebral contusions, which contribute to secondary brain injury, and low GCS scores at day 5 of hospitalization, while not always intervenable, play crucial roles in identifying patients at higher risk of poor neurologic outcomes. Although our findings show that concurrent IVH in patients with DAI was not associated with increased mortality, it was associated with lower HD5GCS, which is a predictor of poor outcomes. These results align with prior research that suggests that the presence of IVH is associated with severe DAI.^32^

This study also highlighted that patients who received blood products, vasopressors, or hyperosmolar therapy in the first 24 hours of admission had a higher likelihood of a low GCS score at hospital day 5. This follows clinical reasoning in suggesting that these therapies and interventions are more common among patients with more severe impairment in neurological function who are at higher risk for poorer outcomes, although based on our findings we cannot conclude whether these therapies themselves may be a driver of poor outcomes. The presence of IVH was also associated with a higher likelihood of a lower GCS score, further emphasizing the impact of concurrent IVH on neurological impairment. In contrast, the use of antiepileptic drugs (AED) was associated with a higher likelihood of achieving a higher GCS score on hospital day 5, suggesting a potential beneficial effect of AEDs in preserving neurological function. Within this context, it is interesting to note that there was no significant association between seizure during admission and HD5GCS. Further studies are needed to establish a conclusive association between AED use and improved GCS scores in DAI patients, and to investigate whether this association varies across different AEDs. (Our institution typically uses a prophylactic regimen of levetiracetam 1.5 grams [g] followed by 1g BID for seven days.) The potential neuroprotective effects of AEDs warrant additional investigation.

## LIMITATIONS

The retrospective design of our study and the reliance on EHR for data collection introduced inherent biases and constraints to accuracy of data collection that we were unable to audit, such as the possibility of manual input errors or inaccurate time measurements. It is important to acknowledge that the collection of blood pressure values in our study lacked standardization. Variations in the type of equipment used, whether invasive or non-invasive readings were employed, and the timing of data collection could have introduced variability into the measured BP values.

As patients with DAI are associated with severe TBI, our population was also likely to have been more critically ill. This is also evidenced by the percentage of our study population requiring mechanical ventilation (92%) and eventual tracheostomy (61%). This may have introduced an indication bias, in which there was closer monitoring and tighter control of blood pressure parameters. Another limitation is that our analysis was conducted at a single center, which is a regional, quaternary trauma and neurotrauma specialty center. This setting potentially limits the generalizability of our findings to patients with DAI presenting in other healthcare settings. Variations in patient characteristics, treatment protocols, and access to resources across different centers may influence the observed associations. Additionally, the relatively small sample size of our study may have limited the statistical power to detect smaller associations between BPV and DAI outcomes.

## CONCLUSION

Patients with radiographically diagnosed diffuse axonal injuries face high rates of morbidity and mortality; only 8% of patients within our study population were discharged home directly from the hospital. Blood pressure variability was not identified as a predictor of discharge disposition. We identified that Glasgow Coma Score at hospital day 5, initial INR, and concurrent cerebral contusion as potential drivers of poor outcomes. It is unclear from our study whether interventions aimed at these variables (eg, correcting an elevated INR) would have affected patients’ outcomes, or whether tight control of patients’ blood pressures with titratable infusions (both antihypertensives and vasopressors) may have masked the impact of BPV on outcomes.
